# Infliximab in Autoimmune Inner Ear Disease: A Case Report

**DOI:** 10.1002/ccr3.70218

**Published:** 2025-02-13

**Authors:** Pauline Millan, Kehinde O. Sunmboye

**Affiliations:** ^1^ Medicine University Hospitals of Leicester NHS Trust Leicester UK; ^2^ Rheumatology University Hospitals of Leicester NHS Trust Leicester UK

**Keywords:** anti‐tumor necrosis factor alpha therapy, autoimmune inner ear disease, biologics, immune‐mediated hearing loss, infliximab

## Abstract

Autoimmune inner ear disease (AIED) is characterized by bilateral, asymmetric, and fluctuating sensorineural hearing loss (SNHL) with no identifiable cause that responds to immunosuppressive therapy. Diagnosis can be challenging due to a lack of standardized diagnostic criteria and pathognomonic tests. The mainstay of treatment is corticosteroids; however, only a small number of patients remain responsive after prolonged use. There are no agreed treatment protocols for AIED following corticosteroids, as there is limited data from randomized controlled trials. We report a case of a 27‐year‐old man with secondary AIED on a background of ulcerative colitis (UC) who experienced frequent relapses and deterioration in his hearing despite multiple courses of high‐dose corticosteroids. He received methotrexate and azathioprine but did not show clinical or audiometric improvement. After the commencement of infliximab infusions, his symptoms of AIED and UC improved, and his hearing remained stable without further use of oral corticosteroid therapy. The available studies on the efficacy of biologic therapy are limited and have produced variable results, with the majority of the data relying mainly on case reports and case series. Large, multicenter randomized controlled trials are required to confirm its efficacy in the management of AIED.

**Summary**

- Autoimmune inner ear disease is a rare but treatable cause of sensorineural hearing loss.
- While corticosteroids remain the mainstay of therapy, the role of corticosteroid‐sparing agents, including biologics, requires further investigation due to limited and variable evidence in the current literature.

## 1 Introduction

Autoimmune inner ear disease (AIED) is a condition first described by McCabe in 1979 based on a diagnostic study on a cohort of patients [1] who presented with bilateral sensorineural hearing loss (SNHL) with a specific clinical pattern that did not fit with existing diagnoses and demonstrated audiometric improvement with corticosteroid and immunosuppressive therapy. The pathogenesis of AIED is not completely understood, though several mechanisms have been proposed, including uncontrolled humoral and cell‐mediated reactions against inner ear antigens [2] resulting in autoantibody development and T‐cell responses [3]. AIED accounts for < 1% of SNHL cases [2] but may be underdiagnosed due to a lack of standardized diagnostic criteria [4]. The main treatment involves the use of corticosteroids; however, the overall reported response is 60%–70% [2] and only 14% remain responsive after 34 months [5]. There is a risk of further decline in hearing in the absence of therapy. Alternative immunosuppressive treatments [3] have been used, but these have yielded variable results. There is no standard treatment protocol for AIED following corticosteroids, as randomized controlled trials are limited [6]. Here, we report a case of AIED in a patient that responded well to infliximab.

## 2 Case History/Examination

**FIGURE 1:**
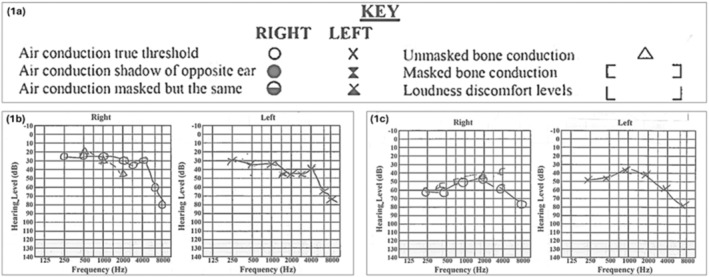
(a) Audiogram key; (b) Audiogram from initial presentation to ED showing mild, symmetrical bilateral hearing loss; (c) Audiogram from second ED presentation after stopping high‐dose steroids showing moderate, symmetrical bilateral hearing loss.

A 27‐year‐old man with a history of ulcerative colitis (UC) diagnosed at 19 years old, migraines, and childhood asthma presented to the emergency department (ED) with a 2‐week history of severe vertigo, vomiting, bilateral hearing loss (left ear worse than the right) and tinnitus. He has a family history of ischemic heart disease. At the time, the patient was on azathioprine 200 mg daily for his UC and had been on the medication for years but had not achieved remission, still having loose stools multiple times per day. On examination by the ENT team, he was noted to have left‐beating nystagmus and catch‐up saccades, and an audiogram showed mild, symmetrical hearing loss (Figure 1a,b). Admission blood tests, including C‐reactive protein (CRP), were unremarkable other than a slightly raised neutrophil count (Table 1). During his 6‐day inpatient admission, an MRI of the head and bilateral internal auditory meatus (IAM) performed with and without contrast showed no mass lesion in the cerebellopontine angle or internal auditory canals, and no cause could be identified for the patient's symptoms.

**TABLE 1:** Blood test results from the patient's first and second ED presentations.

Test	Normal range	Units	First ED presentation	Second ED presentation
WBC	3.6–11.0	×10^9^/L	10.0	10.7
Hemoglobin	130–180 (adult male)	g/L	166	160
Platelets	140–400	×10^9^/L	324	291
Neutrophils	1.8–7.5	×10^9^/L	8.39	9.21
Lymphocytes	1.0–4.0	×10^9^/L	1.02	1.00
Sodium	133–146	mmol/L	139	140
Potassium	3.5–5.3	mmol/L	3.6	4.5
Urea	2.5–7.8	mmol/L	7.4	6.6
Creatinine	59–104 (adult male)	μmol/L	85	81
eGFR	> 90		> 90	> 90
Albumin	35–50	g/L	52	51
ALP	30–130	U/L	85	68
ALT	< 41 (adult male)	U/L	19	40
Bilirubin	< 21	μmol/L	18	9
Amylase	28–100	U/dL	21	
CRP	< 5	mg/L	< 5	15
HbA1c	< 42	mmol/mol		30
TSH	0.4–4.0	mU/L^2^		1.4
Adjusted calcium	2.2–2.6	mmol/L		2.20
Phosphate	0.74–1.4	mmol/L		1.05
Magnesium	0.7–1.0	mmol/L		0.89
Myeloma screen			Negative	
Treponemal antibody			Not detected	
C3	0.75–1.65	g/L		1.50
C4	0.20–0.65	g/L		0.30
Rheumatoid factor	< 20	IU/mL		10
ANA titre				Negative
ANCA (immunofluorescence)				P‐ANCA 1:40
MPO‐ANCA (chemiluminescence)	< 3.5	IU/mL		3.9
PR3‐ANCA (chemiluminescence)	< 2	IU/mL		1.1
ACE	13–64	U/L		23
Anti‐CCP	< 7	U/mL		1

## 3 Differential Diagnosis

The managing team treated the patient for bilateral labyrinthitis with prednisolone 60 mg daily, tapering the dose by 10 mg daily. His symptoms improved, and they discharged him with a follow‐up plan for vestibular rehabilitation and a balance clinic. Less than 2 weeks later, the patient presented to the ED again with a relapse of his symptoms.

A repeat audiogram showed moderate, symmetrical hearing loss (Figure 1c). Routine blood tests showed a mildly raised neutrophil count again but were otherwise unremarkable. Further investigations were conducted, including an autoimmune screen to exclude other systemic autoimmune diseases, myeloma screen, and Treponemal antibody testing, all of which were negative (Table 1).

The patient had treatment with a second course of high‐dose prednisolone 60 mg daily and was discharged home with an urgent follow‐up in the Emergency ENT Clinic. The managing team referred the patient to the Vestibular Rehabilitation Team and the Rheumatology team for suspected AIED. Further management for him included the use of bilateral intratympanic Solu‐Medrone injections by the ENT team and an 8‐week tapering course of prednisolone 40 mg daily. Several tests, including cortical evoked response audiometry which showed that both ears had mid‐frequency hearing loss of around 60 dB and higher frequency hearing loss of over 100 dB. The Rheumatology team reviewed him and confirmed the diagnosis as secondary AIED.

## 4 Conclusion and Results (Outcome and Follow‐Up)

**FIGURE 2:**
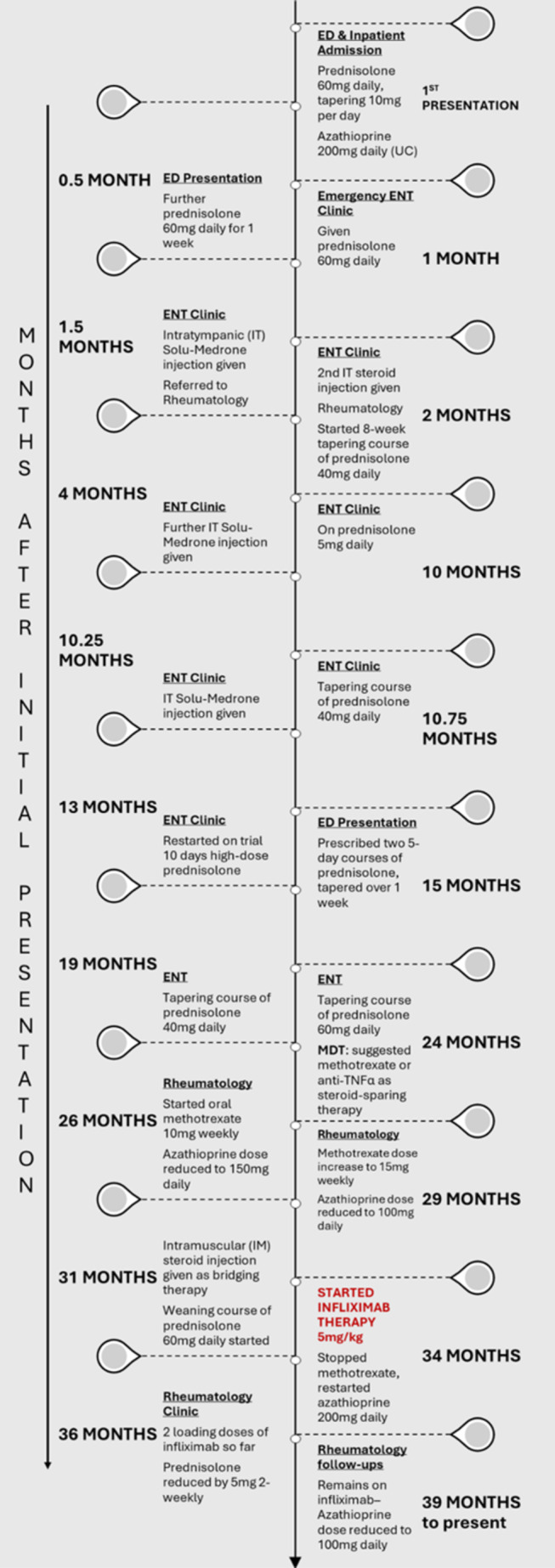
A summary of treatments received.

**FIGURE 3:**
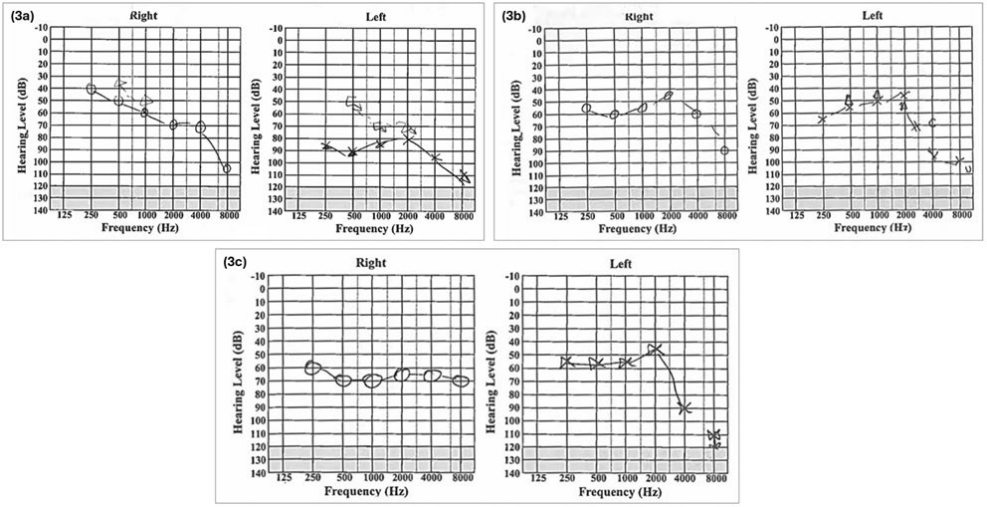
(a) Audiogram during an acute deterioration in hearing. At the time, the patient was on azathioprine 200 mg daily and had completed a weaning course of prednisolone 10 days prior. (b) Audiogram on completion of a weaning course of high‐dose prednisolone 60 mg daily. The patient was on azathioprine 200 mg daily. (c) Baseline audiogram prior to commencing infliximab therapy. At the time, the patient was on azathioprine 150 mg daily, methotrexate 15 mg weekly, and received a STAT dose of intramuscular Depo‐Medrone 120 mg as bridging therapy. He was also commenced on a weaning course of prednisolone 60 mg (reduced by 5 mg every 2 weeks). This is worse than the audiogram from his initial ED presentation (Figure 1b), this has been maintained on infliximab infusions, with no further deterioration whilst remaining off corticosteroids.

Over the span of 2.5 years, the patient received multiple intratympanic steroid injections and prolonged tapering courses of high‐dose prednisolone. Figure 2 shows a summary of the treatments he received and highlights the extensive number of steroid courses he had. Of note, the patient no longer responded to prednisolone 40 mg over the course of his illness, requiring prednisolone at 60 mg daily (tapered over 12 weeks) (Figure 3a,b). While he had clinical and audiometric improvements with steroid therapy, his hearing loss and symptoms would worsen when he was on lower doses or off steroids, with relapses occurring as early as 3 days after discontinuation. This, alongside the fluctuating nature of his symptoms, made his management challenging.

The patient's case was discussed at a multidisciplinary team (MDT) meeting in a specialist center, with an outcome to consider methotrexate or the use of anti‐tumor necrosis factor α (anti‐TNFα) treatment as steroid‐sparing therapy. Simultaneously, the patient was also referred to the local Gastroenterology team to centralize his care and optimize the management of his UC, as he was still passing loose stools multiple times daily. With guidance from the Rheumatology team, he was started on oral methotrexate 10 mg weekly (later increased to 15 mg). He had azathioprine tapering to a dose of 100 mg daily.

His fecal calprotectin levels remained elevated along with clinical symptoms of increased bowel output. The Inflammatory bowel disease (IBD) MDT approved his treatment with infliximab.

The patient was commenced on infliximab infusions 2.5 years after his initial presentation, dosed at 5 mg/kg. He received a loading dose at 0, 2, and 6 weeks, then 8‐weekly maintenance infusions after. Prior to starting infliximab, his methotrexate was stopped, and his azathioprine dose was restored to its original dose of 200 mg daily. He also received a STAT dose of intramuscular Depo‐Medrone 120 mg and a weaning course of prednisolone 60 mg daily as bridging therapy. Once he was started on infliximab, the patient's prednisolone was reduced by 5 mg every 2 weeks until this was stopped. After two loading doses of infliximab, the patient reported that his hearing had stabilized with no further deterioration from his pre‐infliximab baseline audiogram (Figure 3c), along with improvement in his bowel habits and symptoms of UC. The Gastroenterology team reduced his azathioprine dose to 100 mg daily once his infliximab levels were therapeutic. He has remained on this dose in conjunction with regular infliximab infusions.

After a 24‐month follow‐up, reviews by ENT, Rheumatology, and Gastroenterology, the patient's hearing has remained stable (with no further deterioration from the baseline audiogram in Figure 3c) and his UC was in remission. He has not required further courses of corticosteroids since commencing infliximab therapy.

## 5 Discussion

AIED is a rare cause of bilateral SNHL, accounting for < 1% of cases, with a yearly incidence of < 5 per 100,000 [2]. It was described by McCabe in 1979 following a case series involving 18 patients who presented with bilateral SNHL (> 30 dB for at least 3 frequencies) that showed clinical and audiometric improvement to immunosuppressive therapy, with no clear cause [1, 4]. The hearing loss in AIED is characterized by its bilateral, asymmetric, and fluctuating nature [6, 7]. Patients can also report tinnitus and ear fullness (25%–50%), and vestibular symptoms such as disequilibrium, ataxia, nystagmus, and episodic or positional vertigo (50%) [2, 7]. It has a rapidly progressive onset, developing between 3 and 90 days.

The pathogenesis is not completely understood, and studies on AIED have been difficult due to (1) the limited anatomical access to the cochlea, (2) unreliable data from the peripheral blood immune system, and (3) a lack of an ideal animal model [5]. The proposed mechanism for AIED is thought to be an uncontrolled, combined humoral and cell‐mediated reaction against inner ear antigens [2, 3] which promotes autoantibody formation and pro‐inflammatory T‐cell responses. This results in: (1) cochlear vascular injury due to immune complex deposition, (2) autoantibody‐related microthrombosis, and (3) vascular changes involving electrochemical disturbances and impaired neurosignalling [2].

When the inner ear is the only organ affected, AIED is considered ‘primary’. If it occurs with systemic autoimmune diseases such as systemic lupus erythematosus, rheumatoid arthritis and Sjogren's syndrome, it is considered ‘secondary’. Secondary AIED accounts for up to 30% of cases [2]. The Harris AIED classification [7] further categorizes the condition into six different types. Our patient's condition co‐exists with UC and is classified as secondary AIED, or ‘type 2: rapidly progressive bilateral SNHL with systemic autoimmune disease’ [7].

There is a lack of standardized diagnostic criteria and pathognomonic tests for AIED, so diagnosis can be challenging and is often based on exclusion. Diagnosis typically relies on three factors: clinical evaluation, blood tests, and response to corticosteroids [2, 7]. Laboratory tests must be conducted in all patients with suspected AIED. This includes routine blood tests and autoimmune screening (Figure 1) to investigate for underlying systemic autoimmune disease and exclude other differential diagnoses [2]. There are no confirmed serological markers, but a few have been proposed, such as heat shock protein 70 (HSP70) antibody, an autoantibody that targets an inner ear antigen [8]. It was thought to be a predictor of steroid responsiveness, but in a further study, it was found to be insufficiently sensitive to detect AIED and lacked specificity to predict steroid responsiveness [5, 7]. A response to corticosteroids is defined as meeting these two requirements: (1) improvement in SNHL > 15 dB at any frequency OR > 10 dB in at least two consecutive frequencies OR an increase in > 12% in word recognition score (WRS) and (2) no additional pure‐tone air conduction threshold loss at any frequency or decrease in WRS [2, 7]. MRI also plays a role in the diagnosis to rule out retrocochlear pathologies [2].

The clinical features of AIED can mimic other inner ear pathologies such as sudden sensorineural hearing loss (SSHL) and Meniere's disease (MD) [2, 8], and may often be misdiagnosed. It can be differentiated by observing the timing of disease onset; for example, hearing loss in SSHL is characterized by an acute onset of < 3 days and in MD, this typically occurs over years [2]. A case report by Ho et al. [2016] discussed a patient with AIED presenting as MD [8] who, after 4 years of symptom onset, presented with psoriasis, joint pains and anterior uveitis. It was then that a diagnosis of AIED was suspected. The patient was started on a trial of corticosteroids, which he responded to, and was switched to steroid‐sparing therapy [9]. In our patient's case, he was initially diagnosed with bilateral labyrinthitis following his unremarkable MRI and blood test results. The diagnosis of AIED was considered approximately 5 weeks after his initial presentation after he re‐presented multiple times with a deterioration in his hearing shortly after finishing his course of steroids. However, it took almost 1 year from his initial presentation and referral to an ENT specialist that undertook cortical evoked response audiometry to definitively confirm the diagnosis of AIED.

AIED is one of the few forms of SNHL which may be reversible, so prompt medical management is crucial as it may improve the rates of reversing hearing loss [4]. The patients in McCabe's study in 1979 were initially treated with cyclophosphamide and corticosteroids [1] however, due to the substantial risks associated with long‐term cyclophosphamide use, including malignancy and myelosuppression, most clinicians favored high‐dose prednisolone (60 mg daily) as the primary treatment for AIED [6]. High‐dose steroid use is also associated with health risks and adverse effects [6] such as hyperglycaemia, weight gain, and fractures secondary to osteoporosis. A study found that with good patient education and monitoring, it is a safe and effective treatment for AIED [10] but is not effective for long‐lasting management and is associated with episodes of relapse [11].

Although corticosteroids remain the mainstay treatment, there are varying levels of steroid responsiveness initially and throughout the treatment. One study evaluating the efficacy of AIED treatment reported that the overall patient response rate to oral prednisolone was 69.7% [6, 7]. Another study found that only 14% remain corticosteroid responsive after 34 months, most notably in those who present with repetitive deterioration in hearing requiring corticosteroid treatment [6]. Our patient received numerous courses of steroids during this treatment process and initially saw an improvement in his symptoms with prednisolone 40 mg OD but later required 60 mg OD to get the same response. He would also experience a worsening of his symptoms when he is on lower doses or off steroids, relapsing as early as 3 days after stopping treatment.

There is a risk of further decline in hearing in the absence of therapy, and yet there is no standard treatment protocol for AIED following corticosteroids, as randomized controlled trials (RCTs) are limited [7]. Studies regarding alternative steroid‐sparing therapies have yielded variable results, with much of the data relying on case reports and case series [3, 11]. Alternative therapies that have been trialed include methotrexate, azathioprine, and immunomodulators [11] such as biologics, including TNFα inhibitors (etanercept and infliximab), CD20 inhibitors (rituximab) and IL‐1 inhibitors (anakinra and canakinumab). If medical treatment fails and hearing is lost, cochlear implantation is an effective treatment option for these patients [4].

The efficacy of methotrexate was assessed in a multicenter clinical trial as a potential steroid‐sparing therapy in corticosteroid‐responsive patients but did not demonstrate a greater effect than the placebo [5]. A case report described a presentation of relapsing AIED in a 35‐year‐old woman who had a significant response to methotrexate and azathioprine dual therapy. This combination yielded positive outcomes and was well tolerated [12]. Our patient was initially tried on methotrexate in combination with azathioprine, which he was already taking for his UC, but unfortunately showed little to no improvement.

Our patient's AIED is classified as secondary and, more specifically, it fits the Harris AIED classification type 2 that often worsens with a flare of the autoimmune condition. This type is steroid‐responsive and can be managed with targeted therapies that are effective against the underlying condition [7]. The AIED specialist center and Rheumatology agreed that his AIED may be a manifestation or complication of UC. This, combined with the failure to achieve remission for his UC despite being on azathioprine and receiving multiple courses of high‐dose steroids, led the MDT to commence him on infliximab. The patient showed clinical and audiometric improvement after just two loading doses of infliximab infusions. He also reported improvement in his bowel habits and symptoms of UC since starting therapy and has managed to achieve remission. It is, therefore, important to consider secondary AIED in patients with a background of UC. The patient has tolerated infliximab well and has remained on the infusions, in conjunction with azathioprine 100 mg daily.

On literature review, there are two similar case reports to our patient's presentation. One reported a case of a 49‐year‐old man with AIED who was responsive to corticosteroids but experienced frequent relapses and progressive deterioration of hearing. He was tried on multiple agents, but these failed to stabilize his symptoms. After receiving infliximab treatment, he was noted to have a sustained improve in his hearing and tinnitus. There was an attempt to discontinue his treatment after 46 weeks, but this led to a rapid relapse of his condition, though his hearing quickly recovered after restarting infliximab [13]. Another case report presented a case of AIED in a patient with Crohn's disease that showed improvement to anti‐TNFα therapy, halting the progression of hearing loss, as well as improving hearing by an average of 15 dB across all frequencies. The patient's hearing remained stable after [14].

A pilot placebo‐controlled study on anti‐TNFα therapy, etanercept, in AIED, however, resulted in a different outcome [15]. This involved 20 patients with AIED in a 12‐week blinded placebo‐controlled RCT of subcutaneous etanercept 25 mg twice weekly. The efficacy of etanercept therapy failed to exceed the placebo response. Another study on eight patients with steroid‐refractory AIED concluded that systemic infliximab therapy did not improve hearing loss [16].

Although studies on biologic therapy have variable results on hearing improvement, they have shown benefits in treating other aural symptoms such as ear fullness, vertigo, and tinnitus [8] with a retrospective study reporting that > 80% of patients demonstrated improvement in these symptoms on treatment with adalimumab and rituximab [7]. Some studies involving methotrexate have also demonstrated a similar result, with one reporting an 80%–100% improvement rate in vestibular symptoms [9]. Steroid‐sparing therapy should therefore not be eliminated based on the lack of improvement in hearing alone.

The disparity in response could potentially be explained by factors such as the timing of the treatment relative to corticosteroid use, the type of anti‐TNFα therapy used, and the route of administration [5]. The later adaptive immune responses in AIED are often influenced by the early expression of cytokines during the innate immune response, such as TNF. Elevated levels of TNF are identified to be predictive of corticosteroid responsiveness in AIED. Experimental observations reveal that peripheral blood immune cells from corticosteroid‐responsive patients release high levels of TNF in vitro, and these are reduced significantly upon administration of dexamethasone. It is possible that the initial use of corticosteroids prior to anti‐TNFα administration causes an excessive reduction of the TNF levels, potentially compromising its effectiveness in placebo‐controlled trials [5].

## 6 Conclusion

AIED is one of the few forms of SNHL for which a treatment is available; therefore, early diagnosis and intervention are important. We report a case of secondary AIED in a patient with a significant response to infliximab therapy. Studies on the use of anti‐TNFα therapy for AIED as a steroid‐sparing alternative are limited and show variable results. To assess and establish the efficacy of anti‐TNFα therapy in the management of AIED, large multicenter randomized controlled trials are required that measure not only improvement in hearing but also other aural symptoms. This, however, may be difficult due to the rarity of the condition and the lack of a standardized diagnostic criteria.

## Author Contributions

**Pauline Millan:** data curation, project administration, resources, software, writing – original draft. **Kehinde O. Sunmboye:** conceptualization, data curation, formal analysis, investigation, methodology, project administration, resources, supervision, writing – original draft, writing – review and editing.

## Consent

Written informed consent was obtained from the patient for the publication of this case report.

## Conflicts of Interest

The authors declare no conflicts of interest.

## Data Availability

The data used to support the findings of this case report are available from the author upon reasonable request.

## References

[1] B. F. McCabe , “Autoimmune Sensorineural Hearing Loss,” Annals of Otology, Rhinology and Laryngology 88, no. 5 (1979): 585–589, 10.1177/000348947908800501. 496191

[2] A. Ciorba , V. Corazzi , C. Bianchini , et al., “Autoimmune Inner Ear Disease (AIED): A Diagnostic Challenge,” International Journal of Immunopathology and Pharmacology 32 (2018): 205873841880868, 10.1177/2058738418808680. PMC6213300 30376736

[3] O. Shamriz , T. Y , and M. Gross , “Autoimmune Inner Ear Disease: Immune Biomarkers, Audiovestibular Aspects, and Therapeutic Modalities of Cogan's Syndrome,” Journal of Immunology Research 2018 (2018): 1498640, 10.1155/2018/1498640. 29850616 PMC5937438

[4] R. S. Oz , M. Gluth , and M. S. Tesher , “Pediatric Autoimmune Inner Ear Disease: A Rare, but Treatable Condition,” Pediatric Annals 48, no. 10 (2019): e391–e394, 10.3928/19382359-20190923-01. 31609997

[5] A. Vambutas and S. Pathak , “AAO: Autoimmune and Autoinflammatory (Disease) in Otology: What Is New in Immune‐Mediated Hearing Loss,” Laryngoscope Investigative Otolaryngology 1, no. 5 (2016): 110–115, 10.1002/lio2.28. 27917401 PMC5113311

[6] D. Strum , S. Kim , T. Shim , and A. Monfared , “An Update on Autoimmune Inner Ear Disease: A Systematic Review of Pharmacotherapy,” American Journal of Otolaryngology 41, no. 1 (2020): 102310, 10.1016/j.amjoto.2019.102310. 31733712

[7] A. J. Matsuoka and J. P. Harris , “Autoimmune Inner Ear Disease: A Retrospective Review of Forty‐Seven Patients,” Audiology and Neuro‐Otology 18, no. 4 (2013): 228–239, 10.1159/000351289. 23817208

[8] H. Sakano and J. P. Harris , “Emerging Options in Immune‐Mediated Hearing Loss,” Laryngoscope Investigative Otolaryngology 4, no. 1 (2018): 102–108, 10.1002/lio2.205. 30828626 PMC6383306

[9] E. C. Ho and G. L. Yao , “Autoimmune Inner Ear Disease Presenting as Menière's Disease,” Journal of Laryngology & Otology 130 (2016): S3–S175, 10.1017/s0022215116005569.

[10] T. H. Alexander , M. H. Weisman , J. M. Derebery , et al., “Safety of High‐Dose Corticosteroids for the Treatment of Autoimmune Inner Ear Disease,” Otology & Neurotology 30, no. 4 (2009): 443–448, 10.1097/MAO.0b013e3181a52773. 19395984

[11] B. Balouch , R. Meehan , A. Suresh , et al., “Use of Biologics for Treatment of Autoimmune Inner Ear Disease,” American Journal of Otolaryngology 43, no. 5 (2022): 103576, 10.1016/j.amjoto.2022.103576. 35963108

[12] K. L. Huang , H. C. Lin , C. D. Lin , and P. C. Wu , “Relapsing Autoimmune Inner Ear Disease With Significant Response to Methotrexate and Azathioprine Combination Therapy: A Case Report and Mini Literature Review,” Medicine 102, no. 23 (2023): e33889, 10.1097/md.0000000000033889. 37335659 PMC10256420

[13] R. L. Heywood , S. Hadavi , S. Donnelly , and N. Patel , “Infliximab for Autoimmune Inner Ear Disease: Case Report and Literature Review,” Journal of Laryngology & Otology 127, no. 11 (2013): 1145–1147, 10.1017/s002221511300217x. 24125068

[14] H. Staecker and P. P. Lefebvre , “Autoimmune Sensorineural Hearing Loss Improved by Tumor Necrosis Factor‐α Blockade: A Case Report,” Acta Oto‐Laryngologica 122, no. 6 (2002): 684–687, 10.1080/000164802320396402. 12403135

[15] S. Cohen , A. Shoup , M. H. Weisman , and J. Harris , “Etanercept Treatment for Autoimmune Inner Ear Disease: Results of a Pilot Placebo‐Controlled Study,” Otology and Neurotology 26, no. 5 (2005): 903–907, 10.1097/01.mao.0000185082.28598.87. 16151336

[16] S. Pathak , E. Goldofsky , E. Vivas , V. R. Bonagura , and A. Vambutas , “IL‐1β Is Overexpressed and Aberrantly Regulated in Corticosteroid Nonresponders With Autoimmune Inner Ear Disease,” Journal of Immunology 186, no. 3 (2011): 1870–1879, 10.4049/jimmunol.1002275. PMC3031454 21199898

